# Genevestigator V3: A Reference Expression Database for the Meta-Analysis of Transcriptomes

**DOI:** 10.1155/2008/420747

**Published:** 2008-07-08

**Authors:** Tomas Hruz, Oliver Laule, Gabor Szabo, Frans Wessendorp, Stefan Bleuler, Lukas Oertle, Peter Widmayer, Wilhelm Gruissem, Philip Zimmermann

**Affiliations:** ^1^Institute of Theoretical Computer Science, ETH Zurich, 8092 Zurich, Switzerland; ^2^Department of Biology, ETH Zurich, 8092 Zurich, Switzerland; ^3^Computer Engineering and Networks Laboratory, ETH Zurich, 8092 Zurich, Switzerland

## Abstract

The Web-based software tool Genevestigator provides powerful tools for biologists to explore gene expression across a wide variety of biological contexts. Its first releases, however, were limited by the scaling ability of the system architecture, multiorganism data storage and analysis capability, and availability of computationally intensive analysis methods. Genevestigator V3 is a novel meta-analysis system resulting from new algorithmic and software development using a client/server architecture, large-scale manual curation and quality control of microarray data for several organisms, and curation of pathway data for mouse and Arabidopsis. In addition to improved querying features, Genevestigator V3 provides new tools to analyze the expression of genes in many different contexts, to identify biomarker genes, to cluster genes into expression modules, and to model expression responses in the context of metabolic and regulatory networks. Being a reference expression database with user-friendly tools, Genevestigator V3 facilitates discovery research and hypothesis validation.

## 1. Background

Systems biology explores interactions between components of a biological system. Frequently, the term *systems biology* is used in the context of mathematical modeling of cellular networks in unicellular organisms or in specific cell types of multicellular organisms. A less prevalent but fundamental approach is the modeling of networks at the organism level by including quantitative information about where, when, and why specific components are active. Dissecting the organism network (in contrast to the cellular network) of multicellular organisms helps to understand mechanisms that drive development, the functions of system components, and regulatory networks that trigger responses to environmental cues or to genetic perturbations.

To build such networks at multicellular scale, both expression data and contextual information have to be analysed simultaneously. Although gene expression data and statistical tools are now widely available [1–4], the simultaneous analysis of hundreds or thousands of gene expression arrays in their biological context remains a major challenge for biologists. Additionally, the contextual information (sample metadata) of published experiments is not readily available in a systematic form, but frequently in plain text descriptions, published articles, or in author's personal notes. Collecting and annotating sample metadata using accepted ontologies and combining them with the large amount of published expression data opens new perspectives in the analysis of transcriptomes across many cell types, tissues, organs, and conditions. Additionally, it allows to create large reference expression databases representing a virtual mirror of life's biological processes.

In [5, 6], we presented a novel approach to assemble public expression data for mouse and Arabidopsis into context-related profiles (metaprofiles) and provided easy online access to this database. The earlier versions of Genevestigator, however, offered only a limited number of analysis tools and the original architecture did not allow it to scale with the rapidly increasing number of users and gene expression information from multiple organisms. We therefore developed Genevestigator V3, which is based on a new framework of the original Genevestigator concept. In particular, we

- curated and quality controlled more than 20,000 Affymetrix expression microarrays from human, mouse, rat, Arabidopsis, and barley,
- manually curated pathway reaction networks for mouse and Arabidopsis (see Table 1),
- developed an architecture to distribute the analysis task between the client and the server such that computationally intensive algorithms can run on the client computer,
- designed a database model which provides high scalability and organism-independent modeling,
- performed major improvements in existing analysis tools,
- implemented a more efficient and flexible workflow including workspace storage and figure export, and
- developed a set of new analysis tools for biomarker identification, clustering, biclustering, and pathway analysis.

## 2. Construction

The large number of simultaneous users, the increasing load of server side data processing, and the need to implement advanced analysis tools required the choice of a technology that shares the load between server and client machines. In fact, some analyses such as the clustering of large data matrices require considerable CPU time, frequently overloading a single machine if multiple clustering jobs are sent to it simultaneously. At the same time, we looked for a technology that minimizes hardware and software adaptations on the user side.

A careful analysis of possible technologies revealed that a combination of Java and relational database (RDB) technologies would represent the optimal combination to handle the complex problem of providing a large centralized database and complex, highly interactive analysis tools to users in the biology community. We therefore verified the penetration of Java (and its various versions) in the Genevestigator user base [7], to be sure that a sufficiently powerful version of Java is installed on most user computers.

**Figure 1:**
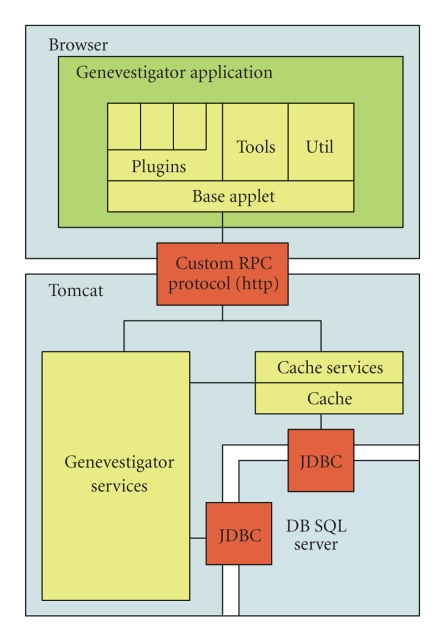
*System architecture of Genevestigator*. The white spaces show the possible boundaries between computers. The application server modules are running in a Tomcat web container and communicate with the database through the JDBC protocol. A thick Java applet reads data from an application server and a cache subsystem through a custom RPC protocol tunneled over http/https. A load-balancing subsystem ensures that the clients can communicate with a scalable cluster of application servers. Moreover, the application servers can be transparently configured to work with a database cluster of an arbitrary size.

Genevestigator V3 comprises four main components: (1) a database, (2) an application server, (3) a Java client application, and (4) a website for user support and management. More specifically, Genevestigator V3 is a multitier Java/Java/Mysql system in which a thick Java client communicates with a farm of server machines running a Tomcat application server which, in turn, communicates with a distributed system of database instances to retrieve the queried data (see Figure 1). Except for a number of specific cases, most of the scientific computing is done on the client side of the application, which provides a highly interactive and integrated view of the analyzed data. This is enabled by Java 2 Swing technology and a custom communication protocol between the Java client and the server farm.

The Genevestigator V3 client application is a signed applet loaded and running within the Internet browser. New versions of the client are distributed on the fly from the server once they are available.

The task of the application server is to cache and quickly provide the compressed data to the client computer, where they could be processed with visualization and clustering algorithms. The application server provides also the data-mining functionality with presampling algorithms to quickly retrieve the data according to biologically meaningful ranking functions.

The database system behind the application server was carefully designed to accommodate multiorganism data and to provide the possibility to switch quickly between different organisms. Another challenge was posed by the experiment classification database subsystem, which stores the measurement data according to ontologies representing the sample experimental context, in particular the anatomy part, developmental stage, stimulus applied, and genetic background. In a further database subsystem, metabolic and regulatory pathways are mapped to multiple organisms and microarray data.

## 3. Content and Tools

In order to build the Genevestigator reference expression database, we collected hundreds of Affymetrix experiments from the public domain, controlled the quality of the data using several Bioconductor packages (see [6]), and manually annotated them according to systematic ontologies. As of March 2008, a compendium of more than 20,000 Affymetrix arrays from human, mouse, rat, Arabidopsis, and barley has been made available for meta-analysis through Genevestigator. This gives users the opportunity to look at how genes are expressed in a very wide variety of contexts. For example, the human dataset currently comprises expression monitoring across more than 330 different conditions, including 89 chemicals, 64 different diseases, 20 growth factors, 17 infection causes, and 36 different tumor types (see Table 1 in Supplementary Material available online at doi:10.1155/2008/420747). An overview of the number of microarrays, pathways, and biological contexts for which expression data is available is shown in Table 1. In Genevestigator, the biological context is embedded into a time-space-response architecture and is given by the terms “Anatomy” (space), “Development” (time), and “Stimulus” or “Mutation” (external or internal perturbations).

**Figure 2:**
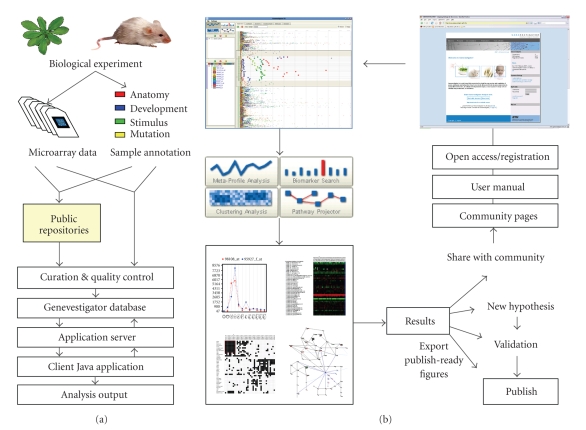
*Data flow and analysis cycle*. Schematic overview of Genevestigator V3. (a) Data flow through the Genevestigator system. Microarray experiments are performed by the research community and either stored in public repositories or sent directly to Genevestigator. Data and annotations are curated and quality controlled by Genevestigator curators and stored in the Genevestigator system (see Figure 1). (b) Expression analysis cycle. The user starts at the Genevestigator website, from where the client application is launched. Four toolsets allow different types of analysis. Results obtained are used either to confirm an existing hypothesis or to create new ones that can be validated in the laboratory. Figures from Genevestigator can be exported in a variety of formats. The website provides user support and a community exchange platform.

An overview of the Genevestigator V3 data- and workflow is shown in Figure 2. Thanks to its novel software architecture and the complete redesign of workflows using usability criteria, Genevestigator V3 allows to work easily with several organisms and multiple lists of genes at the same time. Four toolsets are available to analyze these transcriptomes: (1) Meta-Profile analysis, (2) Biomarker Search, (3) Clustering Analysis, and (4) Pathway Projector. Each toolset comprises several tools that focus on a specific type of analysis. Besides significantly improving the Meta-Profile analysis tools, the Biomarker Search, Clustering Analysis, and Pathway Projector toolsets largely comprise novel tools that were not available in previous Genevestigator releases. More details about these toolsets are provided on the Genevestigator website (https://www.genevestigator.ethz.ch/). In brief;

The *Meta-Profile analysis* toolset includes several tools that provide information about *when*, *where*, and *in response to what* genes of interest are expressed. Specifically, they visualize gene expression across experiments or biological contexts such as anatomy, development, stimulus, and mutation.

The *Biomarker Search* toolset allows users to easily identify genes that are specifically expressed in a set of biological states (anatomy or development) or specifically up- or downregulated in response to a set of perturbations (stimulus or mutation). An explanation on how to use these tools is provided on the website.

The clustering tools in Genevestigator V3 group genes that show similarity in their summarized expression levels (i.e., in their metaprofiles). These tools can also be used to cluster array-level profiles. Two types of methods are currently provided in the *Clustering Analysis* toolset: (1) hierarchical clustering, and (2) biclustering. Both methods have been shown to reveal biologically significant associations between biological components or gene networks [8, 9].

The *Pathway Projector* toolset allows the visualization of expression data on a pathway or network level. Several conceptual features distinguish it from most currently available pathway analysis tools. First, all pathways were verified manually from the literature and modeled into a single reaction network, in which each reaction is represented only once, even if it is shared between several pathways. The user can find locations within the network using classically defined pathway terms. Nevertheless, network analysis focuses on identifying expression modules within the global reaction network, irrespective of classical pathway boundaries. For example, the user can start with a single reaction or pathway and create subnetworks by extending them with neighbor reactions or pathways according to their expression. A second distinguishing feature is the projection of expression metaprofile data onto these networks. The user can create virtually any type of comparison selected from the database and project it onto the network, for instance, compare one state of development with another, a tissue against another tissue, or a treatment against its control set.

## 4. Utility and Discussion

In contrast to the previous versions, Genevestigator V3 integrates all the different analysis tools. For example, marker genes identified using the Biomarker Search tool can be further analyzed by switching to the Meta-Profile or Clustering analysis toolsets. Another major improvement is that all organisms are integrated into the same user client interface, allowing the user to study genes from several organisms in parallel and in the same session.

Besides these analysis tools, several novel usability features have been added to the client, such as saving workspaces, exporting figures in Web or print resolution, saving pathway views, or exporting lists of genes. Users can submit their workspaces or pathway views to be placed on the community page of the Website. This allows other users to view the corresponding results without having to upload arrays or genes again, or to redesign network structures. Additionally, a role-based access control system allows confidential data to be visualized in the context of all other experiments in the database, without being seen by nonauthorized users. Upon request, confidential data from laboratories are quality-controlled and entered into the database by Genevestigator curators to ensure comparability and systematic annotation.

It is generally accepted that the integration of multiple data types will be essential to model biological systems. Transcriptomics data from single experiments provide an informative but selective view of biological processes. There is an increasing evidence, however, that mining microarray data simultaneously from many different experiments and organisms make this type of data highly informative and valuable for functional genomics and systems biology applications. The Genevestigator V3 discovery tool and reference database therefore covers a broad range of academic and commercial research interests. Until now, most researchers have used Genevestigator in molecular biology research for discovery or experimental validation. More recent tools in Genevestigator V3 now allow researchers to develop applications in plant and animal genomics, toxicogenomics, biomarker discovery, crop biotechnology, disease and clinical research, as well as pathway prioritization. Its multiorganism capabilities allow researchers to perform cross-species studies to confirm orthologs and facilitate gene function discovery.

Genevestigator tools are made available in three forms: OPEN-ACCESS, CLASSIC, and ADVANCED. For academic users, the first two are freely available, while access to ADVANCED is made available for a moderate subscription fee (in the range of reagent kits that are frequently used in the lab) to support our costs of curation and development. The tools and features available for each form are described on the Genevestigator website.

## 5. Conclusion

In summary, Genevestigator V3 provides a large and continuously growing reference database of systematically annotated and high-quality microarray data from several organisms. Powerful but user-friendly tools for the meta-analysis of transcriptomes allow to visualize gene expression through a large library of biological contexts, facilitating the modeling of gene regulatory networks and the identification of specific markers. The tool is accessible at http://www.genevestigator.ethz.ch.

## Supplementary Material

The supplementary material provides an example list of all treatments, diseases, and other perturbations mapped within the human datasets in Genevestigator (as of March 2008). Many of them are available from two or more independant studies. This list continues to grow as more data is being curated and made available to the users.

## Figures and Tables

**Table 1:** *Currently available data*. Overview of the number of quality controlled and annotated arrays, partitioned into types of biological contexts for which expression data is available in the database (status: March 2008). For mouse and Arabidopsis, the number of curated pathway reactions is indicated.

	Human			Mouse		Rat		Arabidopsis	Barley
Size of arrays	10 k	20 k	47 k	12 k	40 k	8 k	31 k	22 k	22 k
Number of Affymetrix arrays	1109	3786	2782	3071	1967	2146	858	3110	706
Anatomy									
* * – * * total of nodes in anatomy tree	**97**	**174**	**185**	**150**	**133**	**108**	**63**	**43**	**23**
* * – * * central nervous system	33	46	51	39	16	26	20		
* * – * * cell culture / primary cells	13	39	29	19	21	10	9		
* * – * * other anatomy parts	51	89	104	92	96	69	34		

Development									
* * – * * number of stages available	**9**	**9**	**8**	**12**	**12**	**6**	**7**	**10**	**6**

Stimulus (external perturbations)									
* * – * * total number of treatments	**81**	**295**	**243**	**122**	**81**	**107**	**55**	**168**	**39**
* * – * * chemical treatments	13	97	32	43	34	45	25	40	3
* * – * * diseases, biotic stresses	23	61	34	12	12	4	0	18	24
* * – * * other external perturbations*	45	137	175	67	35	58	30	110	12

Mutation (internal perturbations)									
* * – * * number of mutant models or cell lines	**1**	**4**	**25**	**117**	**100**	**5**	**2**	**167**	**17**

Reaction networks									
* * – * * **Number of reaction nodes**	—	—	—	954	—	—	—	**1199**	—

*includes: surgery, stress, hormone, growth factors and cytokines, exercise, caloric restriction, fasting, peptides, several tumor types, light irradiation, and many more external perturbations.
