# Supplementary material for: Genevestigator V3: A Reference Expression Database for the Meta-Analysis of Transcriptomes
Source: Adv Bioinformatics. 2008 Jul 8;2008:420747. doi: 10.1155/2008/420747 (PMC2777001; doi:10.1155/2008/420747)
Supplement: Supplementary file 1 — The supplementary material provides an example list of all treatments, diseases, and other perturbations mapped within the human datasets in Genevestigator (as of March 2008). Many of them are available from two or more independant studies. This list continues to grow as more data is being curated and made available to the users. [file 420747.f1.pdf]

Supplementary Table 1.

The list below summarizes all treatments, diseases, and other perturbations from the human datasets available for meta-analysis in Genevestigator, as of March 2008. Numbers in brackets indicate the number of independent studies.

Airway brushing (non-smoker)	
Airway brushing (smoker)	
AMD early	(Age-related Macular Degeneration)
AMD late	(Geographic Atrophy)
ALK depletion (shRNA) (2)	(Anaplastic Lymphoma Kinase)
Antibioticum: actinomycin D	
Antibioticum: cycloheximide	
Antibioticum: doxycyclin (4)	
Antibioticum: echinomycin	
Antibioticum: monorden (2)	
Antibioticum: novobiocin	
Antibioticum: trichostatin A	
Antibodies: anti-FcgRIIB (2)	
antiCD3 activation	
antiCD40/Il4 activation	
Beverage intake: grape juice	
Beverage intake: wine	
Beverage intake: alcohol	
Beverage intake: water	
Cardiac allograft rejection	
c-MYC depletion (RNAi) (4)	
Cell cycle inhibition	
Chemical: AG1478	
Chemical: AM580	
Chemical: arachidonic acid	
Chemical: arachidonyltrifluoromethane	
Chemical: arsenite	
Chemical: atorvastatin (2)	
Chemical: atorvastatin / mevalonate (2)	
Chemical: Bay11-7082 (2)	
Chemical: bexarotene	
Chemical: bicalutamide	
Chemical: BPDE	
Chemical: camptothecin	
Chemical: celecoxib	
Chemical: CEP-14083	
Chemical: CEP-14513	
Chemical: clofibrate	
Chemical: chloroguine	
Chemical: chlorpromazine	
Chemical: chlorpropamide	
Chemical: ciclosporin	
Chemical: cisplatin	
Chemical: cisplatin/oxaliplatin	
Chemical: cobalt chloride	
Chemical: copper sulfate	
Chemical: DAPT	
Chemical: dexamethasone (2)	
Chemical: dioxin	
Chemical: exisulind	
Chemical: fludrocortisone	
Chemical: fulvestrant	
Chemical: geldanamycin	
Chemical: gemcitabine	
Chemical: gemcitabine / bexarotene	
Chemical: genistein (2)	

Chemical: haloperidol
Chemical: iloprost
Chemical: imatinib
Chemical: indometacin
Chemical: lipopolysaccharide
Chemical: LM-1685
Chemical: LY294002 (4)
Chemical: menadione (1)
Chemical: metformin
Chemical: metoprolol
Chemical: mevalonate (2)
Chemical: MK886 (3)
Chemical: monastrol (2)
Chemical: motexafin gadolinium
Chemical: nebivolol
Chemical: nifedipine
Chemical: nordihydroguaiaretic acid
Chemical: NU-1025
Chemical: oxaliplatin
Chemical: peginterferon (2)
Chemical: phorbol 12-myristate 13-acetate
Chemical: pirinixic acid (2)
Chemical: PP-2
Chemical: prednisolone (2)
Chemical: resveratrol
Chemical: rofecoxib
Chemical: rosiglitazone (2)
Chemical: rosiglitazone / AGN193109
Chemical: rotenone
Chemical: RTI-6413-018
Chemical: sapphyrin PCI-2050 (2)
Chemical: sapphyrin PCI-5002 (2)
Chemical: sapphyrin PCI-5002 / ZnOAc2
Chemical: sapphyrin PCI-5003
Chemical: SC-58125
Chemical: simvastatin
Chemical: sodium phenylbutyrate
Chemical: sphingosine-1-phosphate
Chemical: staurosporine
Chemical: sulindac
Chemical: tacrolimus
Chemical: tamoxifen (2)
Chemical: tetraethylenepentamine
Chemical: thalidomide
Chemical: thioridazine
Chemical: tretinoin (2)
Chemical: troglitazone
Chemical: urocanic acid
Chemical: U0126
Chemical: valproic acid (3)
Chemical: W-13
Chemical: ZnOAc2
Chemical: 5-aza-2'-deoxycitidine
Chemical: 5-aza-2'-deoxycitidine / TSA
Chemical: 17-allylamino-geldanamycin (2)
Deferoxamine stimulation (2)
Disease: acromegaly
Disease: acute placental malaria
Disease: acute quadriplegic myopathy
Disease: adenomyosis
Disease: age-related macular degeneration (2)
Disease: Alzheimer's disease (5)
Disease: amyotrophic lateral sclerosis

Disease: atopic dermatitis (2)
Disease: atrial fibrillation
Disease: bacterial infectious colitis
Disease: Barrett's esophagus
Disease: Becker muscular dystrophy
Disease: bipolar disorder (2)
Disease: calpainopathy (LGMD2A)
Disease: chronic obstructive pulmonary disease
Disease: chronic placental malaria
Disease: Crohn’s disease (2)
Disease: cystic fibrosis (2)
Disease: dermatomyositis (2)
Disease: diabetes
Disease: Down syndrome (trisomy21) (4)
Disease: Duchenne muscular dystrophy (3)
Disease: dysferlinopathy (LGMD2B)
Disease: Edwards syndrome (trisomy18)
Disease: Emery Dreifuss muscular dystrophy (AD)
Disease: Emery Dreifuss muscular dystrophy (XR)
Disease: emphysema
Disease: endometriosis (2)
Disease: fascioscapulohumeral muscular dystrophy
Diaease: glaucoma
Disease: hereditary gingival fibromatosis
Disease: hereditary spastic paraplegia (SPG4)
Disease: Huntington's disease (9)
Disease: Hutchinson–Gilford progeria syndrome
Disease: Job's syndrome (2)
Disease: juvenile dermatomyositis
Disease: juvenile rheumatoid arthritis (JRA)
Disease: juvenile rheumatoid arthritis pauci
Disease: juvenile rheumatoid arthritis poly
Disease: juvenile spondyloarthropathy
Disease: later developed bronchopulmonary dysplasia
Disease: limb-girdle muscular dystrophie (2I)
Disease: lung tumor (2)
Disease: malaria (2)
Disease: MED: A3243G-MELAS
Disease: MED: A3243G-PEO
Disease: MED: mtDNA "Common"-deletion
Disease: ocular surface disorder
Disease: osteoarthritis (2)
Disease: Parkinson's disease (14)
Disease: Patau syndrome (trisomy13)
Disease: plaque-type psoriasis
Disease: polycystic ovary syndrome (2)
Disease: progressive supranuclear palsy
Disease: psoriasis
Disease: Rett Syndrome
Disease: rheumatoid arthritis (4)
Disease: sarcoidosis
Disease: schizophrenia
Disease: Systemic Onset Juvenile Idiopathic Arthritis (SoJIA)
Disease: spina bifida
Disease: subclinical interstitial fibrosis
Disease: ulcerative colitis (3)
Disease: vulvar intraepithelial neoplasia
Euglycemic clamp
EWS/FLI-1 depletion (shRNA) (2)
Exercise (5)
Exogenous antigens (4)
Growth Factors and Cytokines: G-CSF
Growth Factors and Cytokines: heregulin

Growth Factors and Cytokines: heregulin / Chemical: AG1478
Growth Factors and Cytokines: heregulin / Chemical: U0126
Growth Factors and Cytokines: IFN-a2b
Growth Factors and Cytokines: IFN-g (2)
Growth Factors and Cytokines: IL-1b
Growth Factors and Cytokines: IL-1
Growth Factors and Cytokines: IL-4 / GM-CSF
Growth Factors and Cytokines: IL-6
Growth Factors and Cytokines: IL-19
Growth Factors and Cytokines: IL-20
Growth Factors and Cytokines: IL-22
Growth Factors and Cytokines: IL-24
Growth Factors and Cytokines: IL-26d
Growth Factors and Cytokines: KGF
Growth Factors and Cytokines: SDF (4)
Growth Factors and Cytokines: TGF-b (4)
Growth Factors and Cytokines: TNF-a (3)
Growth Factors and Cytokines: TNF-a / IL-1b / IL-6 / PGE2
Heart failure: chronic
Heart-failure: idiopathic dilated cardiomyopathy (2)
Heart-failure: ischemic cardiomyopathy
Heart-failure: familial cardiomyopathy
Heart-failure: hypertrophic cardiomyopathy
Heart-failure: post-partum cardiomyopathy
Heart-failure: viral cardiomyopathy
Heat shock (2)
HIF-1a depletion (siRNA)
Hormone: angiotensin II
Hormone: dihydrotestosterone (3)
Hormone: testosterone
Hormone: testosterone / anastrozole
Hormone: testosterone / letrozole
Hormone: testosterone / tamoxifen
Hormone: 17alpha-estradiol
Hormone: 17beta-estradiol (7)
Hormone: 17beta-estradiol / cycloheximide
House dust mite
Human AB serum
Hyper-osmotic stimulation
Hypo-osmotic stimulation
Hypoxia (2)
IgG / C3bi stimulation (4)
Inadequate protein intake
Infection: adenovirus (3)
Infection: adenovirus c-Myb
Infection: adenovirus A-Myb
Infection: A. fumigatus
Infection: A. phagocytophilum
Infection: B. burgdorferi
Infection: B. burgdorferi / Growth Factors and Cytokines
Infection: C. albicans (2)
Infection: C. parvum
Infection: Epstein-Barr virus
Infection: HIV (2)
Infection: measles virus
Infection: N. meningitides (3)
Infection: oncolytic herpes simplex virus (4)
Infection: P. aeruginosa (2)
Infection: S. flexneri
Infection: S. pneumoniae (7)
Insulin
Intraluminal pressure
Kidney transplantation (7)

Knee immobilization
KSHV microRNA expression
Left ventricular assist device (3)
Leukotriene D4 stimulation
Lipoprotein stimulation
LMO4 deletion
Low density lipoproteins
LPS/Ifng activation
MeCP2 decoy transfection
Mucociliary differentiation
Muscle disuse atrophy (2)
Myoblast differentiation
Obesity (2)
Optineurin depletion (RNAi)
Ovulation (3)
Ox-Low density lipoproteins
p63 depletion (shRNA)
Pediatric septic shock
Peptide: Ac1-25
Pollutant particle exposure
Polyamide: 1R-Chl
Polyamide: ARE (1)
Polyamide: control (2)
Polyamide: FA1 (2)
Polyamide: FA2
Polyamide: (FRDA) FA1 (2)
Polyamide: (FRDA) FA2
Polyamide: HRE (1)
Polycomb depletion (siRNA)
Protein: Galectin-1
Ramos(pinco BCL6DPEST) (5)
recombinant adenovirus infection: cMyb
recombinant adenovirus infection: vMyb
RENT1/hUPF1 siRNA
RhoGDIbeta depletion (siRNA)
Serum Amyloid A stimulation
Serum deprivation (2)
Severe traumatic injury
Shear stress
Smoking (4)
Social isolation
Solar ultraviolet radiation
Surgery: gastric bypass
TLR2/1L stimulation
Trophoblast conditioned media
Trovafloxacin
Tumors: adenocarcinoma
Tumors: aldosterone-producing adenoma
Tumors: anaplastic large cell lymphoma
Tumors: angioimmunoblastic T-Cell lymphoma
Tumors: Barrett's-associated adenocarcinoma
Tumors: B-cell chronic lymphocytic leukemia (7) (B-CLL)
Tumors: bladder cancer (3)
Tumors: breast cancer (7)
Tumors: cervical squamous cell carcinoma (2)
Tumors: colorectal adenomas (small)
Tumors: colorectal adenomas (large)
Tumors: diffuse large B cell lymphoma (6) (DLBCL)
Tumors: follicular lymphoma
Tumors: hairy cell leukemia (2)
Tumors: HNF1-alpha mutated HCA
Tumors: hypopharyngeal cancer
Tumors: kidney cancer

Tumors: mantle cell lymphoma
Tumors: melanoma
Tumors: metastatic prostate cancer
Tumors: multiple myeloma
Tumors: nephroblastom
Tumors: oral squamous cell carcinoma
Tumors: ovarian tumor (clear cell carcinoma)
Tumors: ovarian tumor (endometrioid) (4)
Tumors: ovarian tumor (mucinous) (2)
Tumors: ovarian tumor (serous) (2)
Tumors: papillary thyroid carcinoma
Tumors: peripheral T-Cell lymphoma/unspecified
Tumors: primary effusion lymphoma
Tumors: primary prostate cancer
Tumors: prostate cancer (2)
Tumors: thyroid cancer (center area)
Tumors: thyroid cancer (invasive area)
Tumors: uterine fibroid (3)
Tumors: Waldenström’s macroglobulinemia (2)
UCA / UVR
UPF1 depletion (siRNA)
UV light
Vitamin D3
